# On Reminder Effects, Drop-Outs and Dominance: Evidence from an Online Experiment on Charitable Giving

**DOI:** 10.1371/journal.pone.0134705

**Published:** 2015-08-07

**Authors:** Axel Sonntag, Daniel John Zizzo

**Affiliations:** 1 Centre of Behavioural and Experimental Social Science and School of Economics, University of East Anglia, Norwich, Norfolk, United Kingdom; 2 Behavioural and Experimental Northeast Cluster and Newcastle University Business School, Newcastle University, Newcastle, Tyne and Wear, United Kingdom; University of Reading, UNITED KINGDOM

## Abstract

We present the results of an experiment that (a) shows the usefulness of screening out drop-outs and (b) tests whether different methods of payment and reminder intervals affect charitable giving. Following a lab session, participants could make online donations to charity for a total duration of three months. Our procedure justifying the exclusion of drop-outs consists in requiring participants to collect payments in person flexibly and as known in advance and as highlighted to them later. Our interpretation is that participants who failed to collect their positive payments under these circumstances are likely not to satisfy dominance. If we restrict the sample to subjects who did not drop out, but not otherwise, reminders significantly increase the overall amount of charitable giving. We also find that weekly reminders are no more effective than monthly reminders in increasing charitable giving, and that, in our three months duration experiment, standing orders do not increase giving relative to one-off donations.

## Introduction

We present an experiment that had two goals: (1) to show the usefulness of screening out drop-outs; (2) to verify the effectiveness of reminders and of standing orders to nudge up charitable giving.

With regards to the first goal, at the end of three months where subjects could engage with the experimental tasks online, they were required to come in person to collect their payments. If there are subjects who cannot bother to do so notwithstanding having clarity that this would be required when they began the experiment, and notwithstanding flexibility in the collection time and date, it is a good sign that they do not care about the incentives provided, and this in turn adds obvious noise to the data. Around 16% of our subjects fell into this category, and, as it will turn out, including them or excluding them does make a difference in the conclusions to be drawn from the experiment. Of course, one could think of cases where subjects could still not make any of the dates even though they thought they would when doing the experiment–for example, a protracted illness. While possible in principle, we received no email from these subjects asking for an alternative payment arrangement because of any such reason, although many fellow co-participants who collected their payments did. There is, of course, a connection between our work and the different and large strand of experimental research that has verified the effects of increasing monetary incentives [1–4].

With regards to the second goal, both casual and research evidence point towards the fact that people pay more attention to things they are reminded of. Products or tasks that people are not reminded of, especially when other products or tasks are heavily advertised, are likely to lose out with respect to attracting people’s attention. Our experiment tests both the effects of reminding people on their opportunity to donate to charity and whether allowing for setting up standing orders changed the donation behavior as opposed to one-off donations.

Although several studies so far have investigated the effect of reminders, to our knowledge no one has investigated the effect of varying time intervals yet. Whereas some studies used reminders as a tool to increase response rates [5,6], others nudged people to take decisions that otherwise would not have been taken at all [7]. In yet another domain, a legal consumer protection context, Garrod et al. [8] suggest that requiring reminders for the end of cool-off periods could help consumers make better use of existing consumer protection instruments. Huck and Rasul [9,10] used a postage reminder (six weeks after the initial invitation to donate was sent) in a field experiment about charitable giving and found a significant reminder effect. Also in a field experiment, Damgaard and Gravert [11] tested the effect of donation deadlines with or without an email reminder. Whereas they did not find any significant effect of different deadlines (3, 10 and 34 days), reminding potential donors of their opportunity to give to a good cause increased the probability of donating. Calzolari and Nardotto [12] used weekly email reminders to successfully nudge people to higher gym visiting frequencies. In a meta-analysis of medical studies, Vervloet et al. [13] find that using electronic reminders such as texts, pagers or specially designed electronic devices improved the adherence to chronic medication. Taubinsky [14] shows how firms can successfully use “reminder advertising” when facing inattentive consumers. Karlan et al. [15] investigated the effectiveness of text messages to increase savings and found that people were more likely to reach their savings goal when being reminded on it regularly.

There also is a common understanding among researchers that sending reminders increases response rates to surveys and questionnaires [6,7,16,17]. Conversely, receiving too many reminders, particularly when they are send in the form of emails, may even back-fire and result in less engagement and attention, i.e. the opposite consequence of what was intended [18,19]. However, we could not find any study that compared the effect of different reminder frequencies. We consider this to be an exercise worthwhile undertaking because it is not clear whether reminding people very frequently increased attention levels or, conversely, might even put people off, because they might feel being ‘spammed’.

A rich literature exists on the effect of using different payment systems on consumer behavior. For example, consumers increased in-store expenditures [20] or bought more unhealthy food [21] or focus on different product attributes [22] when using credit cards as opposed to paying in cash. In the context of charitable donations, Huck and Rasul [10] found that transaction costs regarding the actual method of payment affected the probability of donating. More specifically, providing a prefilled payment form significantly increased the response rate. In a door-to-door solicitation field experiment, Soetevent [23] found that only offering payment by debit card instead of offering only cash payment or both, reduced the participation rates but conditional on participation increased the amount of donations. Furthermore, from a large household survey, Jones and Marriott [24] estimated that those who set up standing orders donate more than those who use payroll deductions. The above research findings and casual evidence that charitable organizations are quite keen on persuading someone to setting up a standing order as opposed to simply receive a one-off donation (of a potentially higher amount), makes it worthwhile investigating whether the method of donating affects the overall amount donated. Charitable organizations could deliberately take advantage of people’s inattention towards an initially set up standing order. We think that additionally exploiting people’s status quo bias [25] is a complement rather than a substitute explanation to inattention.

Our key findings are that, while standing orders in our three months duration experiment did not affect the overall amount donated, having reminders did, but weekly reminders did not help any more than having monthly reminders. The positive effect of reminders can only however be identified once drop-outs are excluded from the sample, and we provide an interpretation of this in terms of Smith’s [26] notion of *dominance*, defined as when “the reward structure dominates any subjective costs (or values) associated with participation in the activities of an experiment”.

The rest of the paper is structured as follows. We first describe the experimental design and results, respectively. We then provide a discussion and conclude.

## Research Design

Although laboratory experiments can, in general, be a very useful tool to identify causal relations of subtle behavioral differences, testing the effect of inattention towards a task such as charitable giving in the lab is very limited. First, participants could potentially be affected by an experimenter demand effect [27] nudging them towards higher donations than they would normally make. Second, the opportunity of donating to charity is very salient in the lab context. In daily life, however, making donations to charitable organizations is only one out of many opportunities one can get involved in and potentially one which is even deemed to have lower priority than many other tasks. That is why, as detailed below, instead of a pure lab experiment, we opted for a combination of a standard lab and an online experiment. Specifically, subjects started from an online questionnaire and a laboratory session; they then had three months where to perform tasks online if so they wished; finally, they had to come in person to receive their payment.

### Experimental design and hypotheses

The experiment had a 3 (reminder frequency: no reminder, monthly or weekly reminders) x 2 (method of donation: standing order or one-off donation) factorial design resulting in a total of 6 treatments (see Table 1).

**Table 1:** Experimental design and numbers of subjects per treatment.

	No reminder	Monthly reminders	Weekly reminders
Standing order	39	38	39
One-off donation	39	39	39

Hypothesis 1: Reminding people about the opportunity to donate to charity is expected to increase their charitable donations compared to the situation of no reminders.

Although hypothesis 1 can straightforwardly be derived from the results of previous studies [9,10], it is less clear whether ‘bombarding’ participants with reminders every week would further increase their contributions or whether they are perceiving such reminders as being spammed [18], resulting in even less attention to the good cause and potentially negative consequences on donation behavior [19]. Thus, we tested the following two contradicting hypotheses:

Hypothesis 2a: Sending weekly reminders leads to higher donations as compared to monthly reminders.

Hypothesis 2b: Sending weekly reminders leads to lower donations as compared to monthly reminders.

Evidence suggests that the method of payment can affect the probability of donating [10] and the amount donated [23]. In contrast to the one-off donation treatments, in the standing order treatments participants could donate to charity by setting up standing orders which deducted the specified amounts from their accounts every month automatically. An example that explains the consequences of both methods of payment is provided later on. As people might forget about their initially set up standing orders (and thereby continue to donate inattentively), we expect the total amounts donated over the full duration of the experiment to be higher in the standing order treatments.

Hypothesis 3: Setting up ‘standing orders’ leads to higher total donations than making ‘one-off donations’.

### Procedures

**Fig 1:**
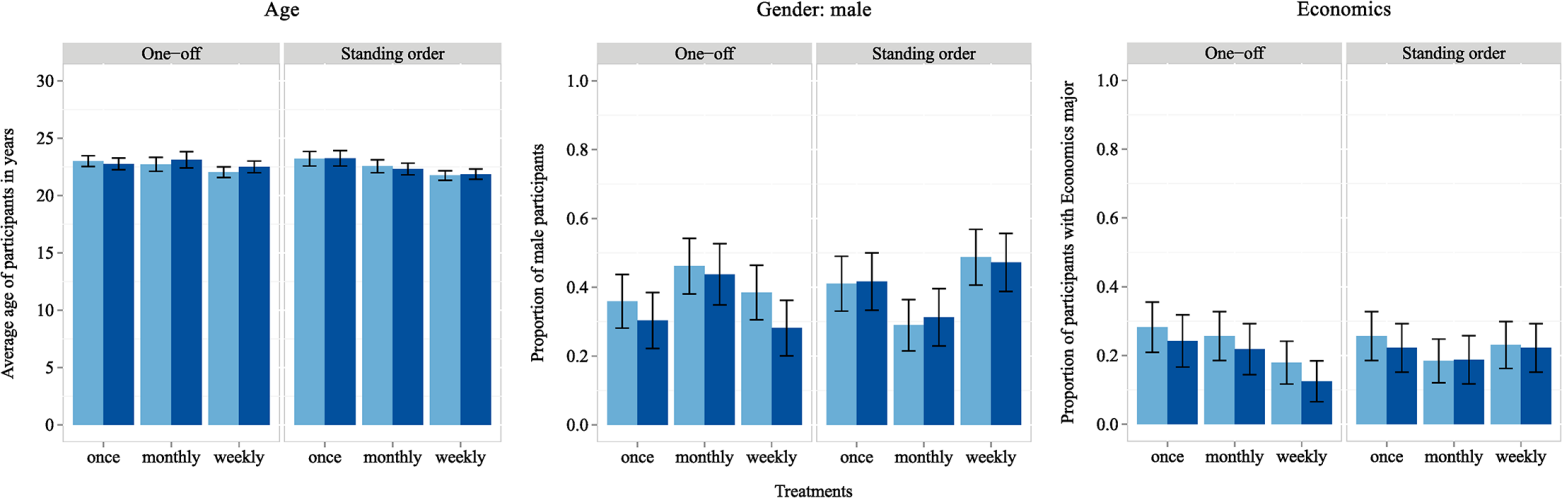
Balance check. Light blue (left) and dark blue (right) bars represent data from the whole sample and the restricted dataset, respectively. Error bars represent standard errors.

Ethical approval was granted by the Research Ethics Committee of the School of Economics at the University of East Anglia. Participants were invited from the CBESS subject pool using ORSEE [28] and provided written consent to take part in the experiment. The subject pool of the Centre for Behavioural and Experimental Social Science (CBESS) contains mainly university students. The sample used for this experiment was well balanced regarding typical demographic dimensions: the average age was 22.5 years (median: 22.0), 39.9% were male and 23.2% had an economics major (see Fig 1). However, in the invitation email they were asked to fill an online questionnaire before coming to the lab session. Only participants who successfully answered the pre-lab questionnaire were admitted to the lab session. Only admitting participants who filled the online questionnaire is unlikely to having caused any sample selection effects as the questionnaire was online for the duration of five days and only very few people were declined to participate in the lab session because they did not fill the questionnaire upfront. The actual lab session consisted of three parts: a standard real effort task, the registration procedure for the experimental online environment and a short questionnaire controlling whether participants really understood the experimental set up. We had a standard real effort task to control for possible house money effects. For example, Reinstein and Riener [29] specifically tested for house money effects in experiments on charitable giving and found that people on average donated less when they had to earn their endowment with a real effort task as opposed to just receiving it by luck. In the standard real effort task participants counted “1s” in a 5x5 matrix [30] and each participant could earn a lump sum of £15, if he/she answered the correct number of “1s” of at least 15 matrices within ten minutes. All participants passed the threshold of 15 tasks. A screenshot of an example task can be found in the S1 File. On average participants spent approximately 45 minutes ‘working’ on the experiment (including the pre-lab questionnaire); i.e., paying £15 plus £2 participation fee results in an hourly wage of £22.7, which is arguably above the average rate of a typical U.K. lab experiment. However, before the participants started the real effort task, we made clear that if they exceeded the threshold they would not be paid directly after the session but would receive a salary of £5 per month for the duration of three months, paid to their personal experimental account. It was also made clear that, in case they did not pass the threshold, they would earn nothing in addition to their £2 participation fee. Participants were informed about the specific payment dates, were trained how to logon to the online experimental portal and how to check their account balances online. Salaries were paid on the 15^th^ of February (first), on the 15^th^ of March (second), and the 15^th^ of April 2013 (third and last). Standing orders were executed one day before the next salary was paid, regardless the day of the week (i.e. standing orders were also executed on Saturdays, Sundays or public holidays). Hence standing order payments were deducted on March 14^th^, April 14^th^, and May 14^th^ 2013. Furthermore, they were informed that they could use their salaries to make donations to Oxfam, an international charity against poverty, also via the online experimental portal. During the lab sessions participants were linked to the website of Oxfam and had the opportunity to browse this site for as long as they wished to gather information about the organization they could donate to in this experiment. After answering a short questionnaire, checking whether participants really understood the experimental set up, participants were paid a participation fee of £2 and left the lab. The check for understanding contained questions about how to access the experimental website, what subjects could do on the website, how much they could earn, what Oxfam is about, how any donations made will be passed on to Oxfam, how the experimental earnings were computed and when they could be collected. All experimental materials, i.e. all questionnaires and instructions, are provided in the S1 File.

During the following three months participants, depending on the treatment they were in, received no, monthly or weekly emails in which we reminded them of their opportunity to check their account balance and to donate to charity by logging on to the online experimental portal. Disregarding that calendar months differ in days, ‘monthly’ reminders were sent every four weeks on February 18^th^, March 18^th^ and April 15^th^ 2013. Weekly reminders were sent every Monday, irrespective how many weeks a month had. Hence, weekly reminders were sent on February 18^th^, 25^th^, March 4^th^, 11^th^, 18^th^, 25^th^, April 1^st^, 8^th^, 15^th^, 22^nd^, 29^th^ and May 6^th^ and 13^th^ 2013 resulting in 13 weekly reminders. The precise texts used in the reminder emails as well as further details on the used procedures can be found in the S1 File. Whereas setting up a standing order had ongoing consequences, making a one-off donation did not. Let us provide an example how the two different methods of payment worked in practice. For instance, assume that a participant in a standing order treatment set up a standing order of £2 in the first month. If this participant did not log on to the online platform later on in the experiment, she would have received a monthly salary of £5 and £2 were deducted from her account every month. In total, she would have earned 3x£5 = £15 and would have donated 3x£2 = £6, thus would have received £15-£6 = £9 on payment day. Standing orders could be changed at any time. A previously set standing order could be revoked by changing its amount to zero. Conversely, if a participant in a one-off donation treatment made a one-off donation of £2 in the first month and did not interact with the online platform later on in the experiment, he would have earned 3x£5 = £15 and would have donated £2, thus would have received £15-£2 = £13.

**Fig 2:**
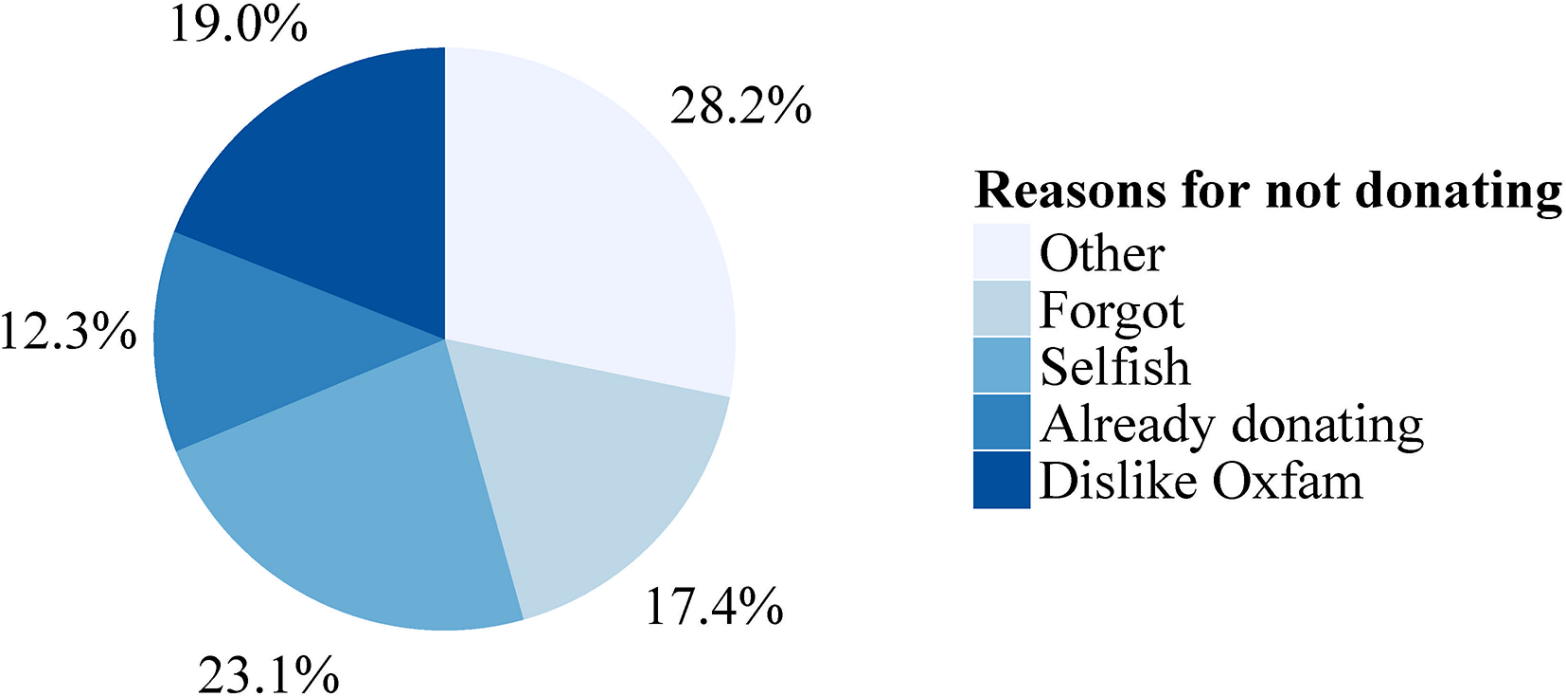
Reasons stated for not donating. Every subject who collected his/her payment filled brief questionnaire in which they explained their main reasons why they did not donate (more). Subsequently these open answers were categorized by the experimenter into five categories (N = 195).

After three months, at the end of the experiment, donated amounts were given to Oxfam, and all participants received an email reminding them that they had to come to the lab one more time to collect their final payment. Before being paid participants were asked to answer a very brief final questionnaire. This questionnaire contained a manipulations check (i.e. how often participants received email reminders, if any), how often they checked their emails during the Easter break, how much they remember having donated and an open question about their motives for (not) donating (see Fig 2). The main payment day was May 15^th^ 2013; however, participants could collect their payments up to four weeks after this date and knew that from the very beginning of the experiment, and arrangements were made flexibly with students to collect their earnings over this period. In total 47 participants (i.e. 20.2% of all participants) sent us emails asking for individual arrangements for collecting their payment on a different day than the main payment day. The S1 File contains further details and a typical example of a related email exchange. This feature is crucial to our design regarding the screening for drop-outs as it minimizes the chance that any participants did not collect their payment because they were not able to either come on the payment day or arrange some individual time and date for payment. In the following we shall refer to the dataset that excludes drop-outs as the *restricted dataset*.

Being fully aware of the potential difficulties in understanding, and to avoid misperceptions, we took extra care to explain the set-up of the experiment to participants. In particular, we made clear the 3- months duration of the experiment and that participants would receive their payments after this time span was stressed three times before participants even started the real effort task (in the invitation email, in the consent form that was signed at the point of entering the lab and in the instructions that were presented to the participants during the lab session). Furthermore, the structure of the experiment was explained in full detail during the lab session and participants could only progress once they completed an extensive check for understanding in which we–again–focused on the structure of the experiment (3 months), the possible actions participants could take (check their balance, donate or do nothing) and how the money would actually be donated to the charity.

## Results

### Collection of payments and drop-outs

In total 233 subjects participated in the experiment, of which 195 collected their payment after the entire duration of three months. Participants were almost equally distributed across treatments resulting in 38 subjects in the MR treatment and 39 subjects in all the other treatments (see Table 1). Participants were also well-balanced across treatments. Performing Kruskal-Wallis tests to check for varying distributions between treatments along the three prominent demographic dimensions displayed in Fig 1 we found no statistically significant difference (p = 0.230, p = 0.535 and p = 0.864 for age, gender and study major economics, respectively). Of the 38 non-collectors, six donated their full earnings of £15 and therefore had no reason to come to collect any payment and cannot properly be called drop-outs from the experiments, in the sense that for them the experiment was concluded with their full donations.

**Fig 3:**
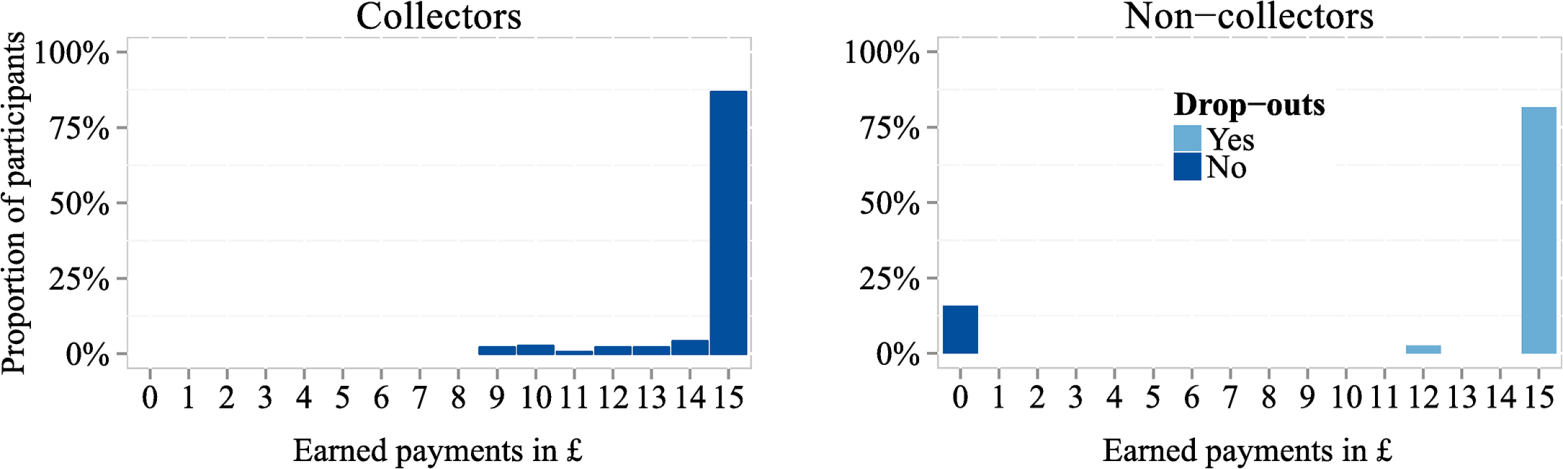
Histograms of earned payments by collectors and non-collectors. The left and the right part contain the observations of participants who collected and did not collect their payments, respectively (n = 195 and 38, respectively).

The remaining 32 out of 233 subjects–that is, 16% of our sample–, did not collect their earnings even though they were entitled to average payments of £14.91. Fig 3 has a histogram of due experimental payments of collectors and non-collectors. These drop-outs gave up at least £12 and on average of £14.91, suggesting that they were insufficiently motivated to engage in the experiment in a way that can provide interpretable data. We shall return to this in section 4. Note that non-collectors donated about 5% of their salary less (Wilcoxon test: p = 0.054), were 12.8% less likely to make a donation (p = 0.055) and donated 0.16 times less often (p = 0.061) than collectors.

In what follows we shall consider our analysis both for the full dataset and the *restricted sample* that excludes the 32 drop-outs that are not likely to be properly incentivized.

### Key results on charitable giving

Table 2 contains some descriptive statistics for the share of salary donated (that is, the total amount donated divided by 15), the probability of making a donation and the number of donation actions. Whereas in the one-off treatments taking a donation action was equivalent to making a donation, in the standing order treatments a donation action refers to the set-up or change of a standing order.

**Table 2:** Descriptive Statistics.

	Method of donation	One-off donation			Standing order			
	Reminder intensity	None	Monthly	Weekly	None	Monthly	Weekly	overall
All observations	Share of salary donated	0.041 (0.169)	0.043 (0.164)	0.039 (0.168)	0.034 (0.106)	0.039 (0.105)	0.103 (0.274)	0.050 (0.174)
	Probability of making a donation	0.103 (0.307)	0.179 (0.389)	0.103 (0.307)	0.103 (0.307)	0.132 (0.343)	0.231 (0.427)	0.142 (0.349)
	Number of donation actions	0.179 (0.601)	0.256 (0.637)	0.103 (0.307)	0.128 (0.409)	0.158 (0.437)	0.385 (0.877)	0.202 (0.578)
Restricted dataset	Share of salary donated	0.042 (0.182)	0.052 (0.180)	0.048 (0.185)	0.037 (0.110)	0.046 (0.113)	0.111 (0.284)	0.057 (0.186)
	Probability of making a donation	0.091 (0.292)	0.219 (0.420)	0.125 (0.336)	0.111 (0.319)	0.156 (0.369)	0.250 (0.439)	0.159 (0.367)
	Number of donation actions	0.152 (0.566)	0.312 (0.693)	0.125 (0.336)	0.139 (0.424)	0.188 (0.471)	0.417 (0.906)	0.224 (0.604)

Means, standard deviations in parentheses

Result 1: The amounts donated increased when monthly reminders were sent instead of sending no reminders at all. This increase is not significant for the full dataset, but is significant for the restricted dataset.

The regression analysis in Table 3 shows that participants in the restricted sample donated over 40% of their salary more when receiving monthly reminders as compared to receiving no reminders at all. This supports hypothesis 1 in relation to the restricted dataset.

**Table 3:** Share of salary donated.

	All observations			Restricted dataset		
	(1)	(2)	(3)	(4)	(5)	(6)
Standing order	0.108	0.098	0.061	0.078	0.038	-0.006
	(0.163)	(0.162)	(0.158)	(0.165)	(0.162)	(0.155)
Monthly reminder	0.261	0.262	0.274	0.401*	0.412*	0.480**
	(0.212)	(0.210)	(0.210)	(0.223)	(0.218)	(0.221)
Weekly reminder	0.369*	0.352*	0.397*	0.477**	0.424**	0.546**
	(0.213)	(0.212)	(0.216)	(0.221)	(0.213)	(0.225)
'Selfish motives'	-1.005***	-1.030***	-1.125***	-1.121***	-1.202***	-1.374***
	(0.371)	(0.373)	(0.395)	(0.367)	(0.369)	(0.400)
'Already donating'	-0.862**	-0.892**	-1.043***	-1.001***	-1.090***	-1.467***
	(0.390)	(0.391)	(0.398)	(0.382)	(0.382)	(0.426)
'No money for Oxfam'	-0.756**	-0.782***	-0.717**	-0.890***	-0.971***	-0.939***
	(0.299)	(0.301)	(0.290)	(0.297)	(0.298)	(0.293)
'Forgot to donate'		-0.150	-0.181		-0.374*	-0.407**
		(0.214)	(0.210)		(0.210)	(0.202)
Organised person			-0.007			-0.015
			(0.058)			(0.057)
Busy person			0.038			0.036
			(0.068)			(0.070)
Emails per day			0.007			0.004
			(0.006)			(0.005)
Procrastination			-0.008			0.001
			(0.010)			(0.011)
Social Desirability			-0.063**			-0.062**
			(0.031)			(0.029)
Age			0.010			0.007
			(0.024)			(0.024)
Male			-0.097			0.089
			(0.184)			(0.186)
Economics			-0.028			-0.064
			(0.185)			(0.192)
Chinese			0.158			0.141
			(0.231)			(0.216)
British			0.189			0.404*
			(0.231)			(0.230)
Constant	-0.825***	-0.772***	-0.517	-0.705***	-0.537**	-0.473
	(0.235)	(0.240)	(0.867)	(0.230)	(0.225)	(0.894)
Observations	233	233	233	201	201	201
Log lik.	-87.743	-87.494	-83.542	-76.187	-74.499	-68.103
Chi-squared	26.662	27.160	35.064	36.749	40.126	52.917
Left-censored (at 0)	200	200	200	169	169	169
Right-censored (at 1)	6	6	6	6	6	6
Uncensored	27	27	27	26	26	26

Table 3 contains coefficients (standard errors in parentheses) of a Tobit model on the share of the salary donated per participant (censoring at 0 and 1). Columns 1, 2 and 3 were estimated using all observations (n = 233) and columns 4, 5 and 6 were estimated with the restricted sample (n = 201). The variable names in single quotes represent encoded dummies that are mutually exclusively 1 if the stated main reason for not-donating matches the dummy’s name and 0 otherwise; ‘Other reasons’ were defined as the base-category. Organized/Busy person: self-perception measured with a 7-point Likert scale, Emails per day: number of emails subjects stated they received each day. Procrastination and Social Desirability were measured using standard psychological scales. Interaction terms of standing order and weekly/monthly reminders (results not included above) were never significant; Levels of significance

***p < 0.01

**p < 0.05

*p < 0.1.

**Fig 4:**
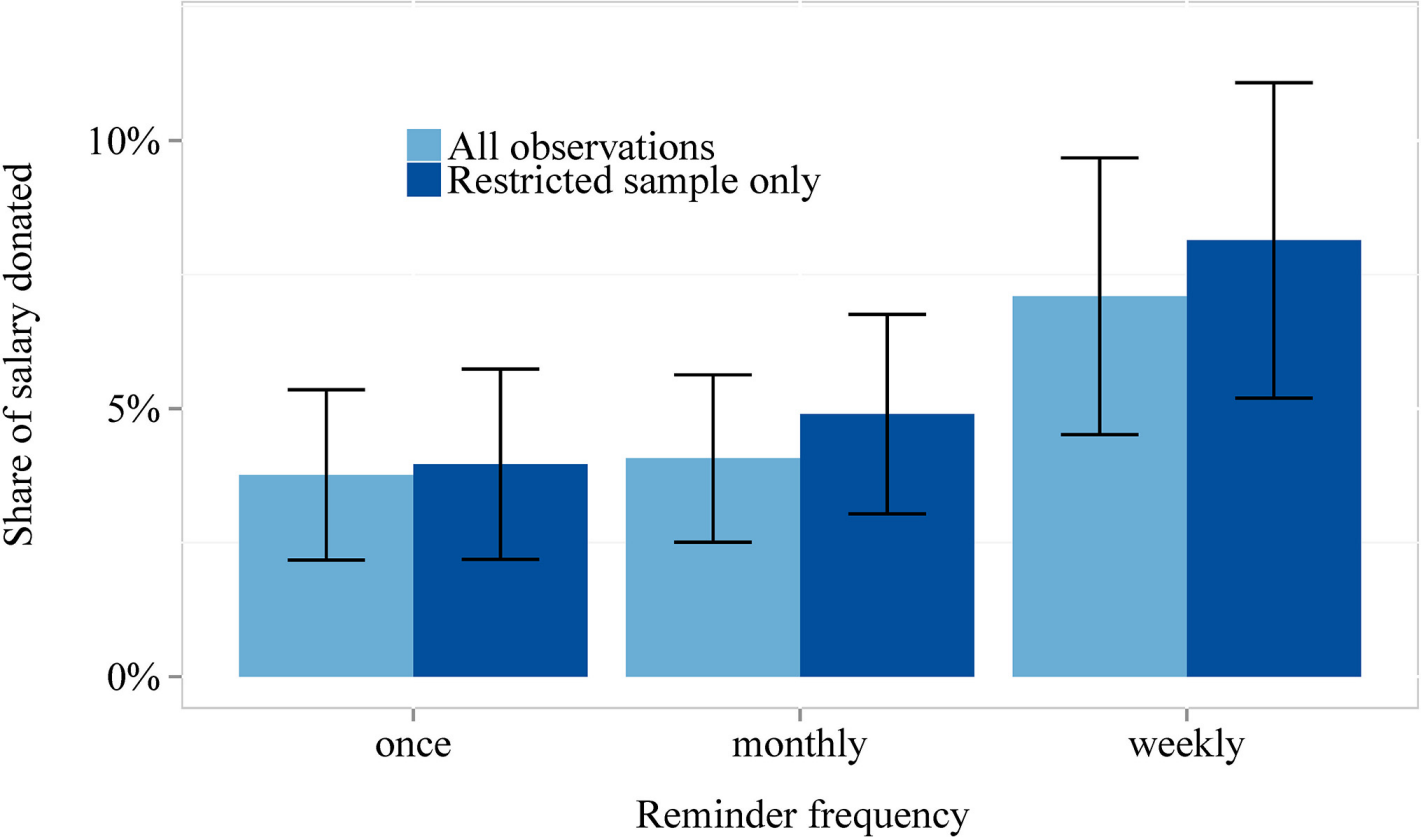
Share of salary donated. Error bars represent standard errors.

As it appears evident from Fig 4, weekly reminders seemed to have an even stronger effect than monthly reminders (participants donated at least 42% of their salary more); however, this increase is not significant compared to monthly reminders (Wald test: p = 0.695, p = 0.952 and p = 0.721 for specification 4, 5 and 6 in Table 3, respectively. In further regressions we had interaction terms with standing orders but did not find them significant.). We therefore do not find support for either hypothesis 2a or 2b.

Result 2: Sending weekly as opposed to monthly reminders did not affect the amounts donated in our three months duration experiment.

Participants who stated (in the final questionnaire) that they were short on money and needed to take care of their own finances first before donating to charity (‘selfish motive’), that they were ‘already donating’ to another charity or that they would not be willing to donate to Oxfam in particular, had donated much less than their co-participants.

The regressions in Table 3 also show that the coefficient on the standing order dummy is small and not significant. Against hypothesis 3, in our three months duration experiment, we find no support for the use of standing orders in place of one-off donations to increase total donations.

Result 3: While the average donation per participant in the standing order treatments was slightly larger than with one-off donations, the difference is not statistically significant.

### Supplementary analysis

#### Probability of making a donation and number of donation actions

The regressions in Tables 4 and 5 show that there are only very weak reminder effects (both in magnitude and statistical significance) in the full dataset for either the probability of making a donation or the number of donation actions. In the restricted dataset, the effect of monthly reminders seems to operate both by increasing the probability of donating and the number of donation actions. Weekly reminders rather only seem to affect the probability of making a donation. However, the effects of monthly and weekly reminders are not significantly different from one another in relation to both the probability of making a donation (Wald test: p = 0.971, p = 0.790 and p = 0.947 for regression 4, 5 and 6 of Table 4, respectively.) and the number of donation actions (Wald test: p = 0.509, p = 0.471 and p = 0.659 for regression 4, 5 and 6 of Table 5, respectively.). Whether weekly or monthly, reminders increase the probability of a donation by around 10%, and the number of donation actions by around 1 on average.

**Table 4:** Probability of making a donation.

	All observations			Restricted dataset		
	(1)	(2)	(3)	(4)	(5)	(6)
Standing order	0.026	0.026	0.022	0.022	0.016	0.012
	(0.039)	(0.039)	(0.038)	(0.042)	(0.042)	(0.036)
Monthly reminder	0.072	0.072	0.072	0.118**	0.121**	0.124**
	(0.049)	(0.049)	(0.049)	(0.053)	(0.053)	(0.049)
Weekly reminder	0.081	0.080	0.090*	0.116**	0.108**	0.127**
	(0.050)	(0.050)	(0.050)	(0.054)	(0.054)	(0.052)
'Selfish motives'	-0.228***	-0.229***	-0.251***	-0.268***	-0.285***	-0.306***
	(0.069)	(0.070)	(0.072)	(0.069)	(0.071)	(0.071)
'Already donating'	-0.192**	-0.193**	-0.226***	-0.238***	-0.256***	-0.325***
	(0.084)	(0.084)	(0.085)	(0.085)	(0.087)	(0.093)
'No money for Oxfam'	-0.172***	-0.173***	-0.163**	-0.215***	-0.232***	-0.215***
	(0.064)	(0.064)	(0.064)	(0.066)	(0.068)	(0.067)
'Forgot to donate'		-0.005	-0.006		-0.064	-0.060
		(0.051)	(0.051)		(0.054)	(0.047)
Organised person			-0.001			-0.003
			(0.014)			(0.013)
Busy person			0.012			0.012
			(0.016)			(0.016)
Emails per day			0.001			0.000
			(0.001)			(0.001)
Procrastination			-0.001			0.001
			(0.002)			(0.002)
Social Desirability			-0.010			-0.010
			(0.007)			(0.007)
Age			0.005			0.005
			(0.006)			(0.006)
Male			-0.008			0.043
			(0.044)			(0.042)
Economics			-0.025			-0.044
			(0.046)			(0.046)
Chinese			0.028			0.021
			(0.055)			(0.049)
British			0.041			0.095*
			(0.056)			(0.054)
Observations	233	233	233	201	201	201
Log lik.	-82.609	-82.605	-79.917	-70.284	-69.539	-63.708
Chi-squared	24.87	24.88	30.25	35.65	37.14	48.80

Table 4 marginal effects of a Probit regression on the probability of making a donation (1 if at least one donation was made, 0 otherwise). Columns 1, 2 and 3 were estimated using all observations (n = 233) and columns 4, 5 and 6 were estimated with the restricted sample (n = 201). Interaction terms of standing order and weekly/monthly reminders (results not included above) were never significant. Levels of significance

***p < 0.01

**p < 0.05

*p < 0.1.

**Table 5:** Number of donation actions.

	All observations			Restricted dataset		
	(1)	(2)	(3)	(4)	(5)	(6)
Standing order	0.297	0.290	0.229	0.237	0.163	0.078
	(0.481)	(0.482)	(0.484)	(0.467)	(0.467)	(0.461)
Monthly reminder	0.832	0.834	0.879	1.270**	1.303**	1.528**
	(0.621)	(0.621)	(0.639)	(0.625)	(0.623)	(0.647)
Weekly reminder	0.948	0.935	1.104*	1.263**	1.173*	1.564**
	(0.621)	(0.624)	(0.653)	(0.616)	(0.612)	(0.655)
'Selfish motives'	-2.923***	-2.950***	-3.343***	-3.154***	-3.351***	-4.007***
	(1.060)	(1.070)	(1.185)	(1.000)	(1.022)	(1.158)
'Already donating'	-2.457**	-2.486**	-2.941**	-2.779***	-2.989***	-4.136***
	(1.121)	(1.132)	(1.176)	(1.047)	(1.067)	(1.198)
'No money for Oxfam'	-1.995**	-2.020**	-1.938**	-2.310***	-2.501***	-2.580***
	(0.833)	(0.844)	(0.846)	(0.787)	(0.806)	(0.821)
'Forgot to donate'		-0.125	-0.161		-0.734	-0.791
		(0.630)	(0.641)		(0.599)	(0.589)
Organised person			0.004			-0.021
			(0.180)			(0.172)
Busy person			0.124			0.109
			(0.209)			(0.204)
Emails per day			0.006			-0.003
			(0.018)			(0.016)
Procrastination			-0.016			0.010
			(0.031)			(0.032)
Social Desirability			-0.138			-0.126
			(0.092)			(0.084)
Age			0.058			0.053
			(0.076)			(0.072)
Male			-0.116			0.478
			(0.562)			(0.547)
Economics			-0.057			-0.251
			(0.570)			(0.577)
Chinese			0.277			0.207
			(0.697)			(0.634)
British			0.539			1.174*
			(0.708)			(0.676)
Constant	-2.389***	-2.349***	-2.851	-1.996***	-1.692**	-2.766
	(0.673)	(0.697)	(2.696)	(0.636)	(0.651)	(2.696)
Observations	233	233	233	201	201	201
Log lik.	-124.933	-124.913	-122.410	-110.994	-110.214	-104.806
Chi-squared	24.560	24.599	29.605	35.094	36.654	47.470
Left-censored (at 0)	200	200	200	169	169	169
Uncensored	33	33	33	32	32	32

Table 5 contains coefficients of a Tobit model on the number donation actions taken per participant (left-censoring at 0), respectively. Columns 1, 2 and 3 were estimated using all observations (n = 233) and columns 4, 5 and 6 were estimated with the restricted sample (n = 201). Interaction terms of standing order and weekly/monthly reminders (results not included above) were never significant. Levels of significance

***p < 0.01

**p < 0.05

*p < 0.1.

The regressions in Tables 4 and 5 also show that, in both the full and restricted dataset, whether payments were made by setting up a standing order or by one-off donations did not affect either the probability of making a donation or the number of donation actions.

Similarly to the regressions on the amount donated, personal factors played a significant role in determining the probability of making a donation or the number of donation actions. For example, participants who stated (in the final questionnaire) that they already regularly gave to charity were less likely to donate to Oxfam via our experiment. Moreover, participants took significantly fewer donation actions if they stated they needed the money for themselves (‘selfish motives’).

#### Power analysis

One might argue that the reason why we did not find significant differences along the standing order treatment dimension was because of a lack of statistical power. In order to address this issue we conducted a post-hoc power analysis to check whether e.g. doubling or quadrupling the sample size would have resulted in higher significance levels. We used the software G*Power 3.1 [31]. Given our observed data, in our three months duration experiment we would have needed over 5300, 9100 and 9100 subjects to participate in this experiment in order to find statistically significant differences between the standing orders or one-off donations for the dimensions of share of salary donated, the probability of making a donation and the number of donation actions, respectively. The above sample sizes represent requirements for treatment differences in the whole sample. The minimum required sample sizes for participants who do not drop out are 5800, 8800 and 6700. It is therefore clear that getting higher significance levels was not just a matter of merely doubling or quadrupling the sample size, but that our data seemed to be rather robust against increasing the sample size to a magnitude that has been used in field experimental settings [32].

## Discussion

We organize our discussion around the two contributions of this paper: using drop-outs as a useful methodological tool, and attempting to increase charitable giving (either by changing the frequency of reminders or by having standing orders).

### Drop-outs and dominance

While in our 3 months duration experiment there is clearly no effect of standing orders no matter the sample, we can identify an effect of reminders, but only when we focus on what we have labeled the restricted dataset, which includes 84% of the observations. Our procedure of removing drop-outs from the sample is justified by the assumption that drop-outs add noise to the data. One interpretation for this is that the subjects who drop out do not sufficiently care about the financial incentives offered in this experiment. Internal validity is lost if there are subjects who clearly do not care about the incentives provided, and our interpretation is that this is what our procedure helps us identify, at least as a first approximation.

Had there been, for example, subjects who donated almost all of their money and did not collect the small remainder, the question could have been asked about whether the reason they did not show up was because of the small size of the remaining money relative to the hassle of coming to collect it, rather than because they did not take the experiment seriously. In our experiment, we did not have such cases (see Fig 3), and so this is not a problem, but it could in principle be an issue in other experiments.

Another potential problem for our interpretation of drop-outs could be that non-collectors did not show up because of last minute problems. We received no email to suggest that this was the case in our experiment, although about 20% of all participants sent emails regarding the arrangements of individualized payment times and dates or if they could send a friend to collect their payment on their behalf, which suggests that, in general, participants were not shy to contact the experimenters in such matters. Furthermore, although last official date for collecting any payment was four weeks after the main payment day, we ‘kept the line open’ (i.e. regularly checked the email address used for this experiment) for an additional 6 months, to make sure we would not lose any subjects who–for any reason–were not able or willing to contact us in time. Any future implementation of this procedure needs to ensure that an email address or equivalent is available for people with last minute problems to contact the experimenters.

An additional objection to validity of our screening procedure could be that participants–quite rationally–did not collect their payments because their travel costs (to university) exceeded their earnings from the experiment. Using the participants’ nationalities as a proxy for potential travel costs (in case students went back to their home countries during the payment period), and comparing the restricted sample proportions across nationalities, we did not find evidence for the travel cost argument in our data. British nationals, who on average should have lower travel costs, appear to be slightly (though not significantly) *more* likely to drop out (81%) than participants with other nationalities (89%).

Our interpretation and procedure justifying the removal of drop-outs requires that subjects know at the beginning that payment will take place in a given time period; that researchers provide flexibility in the payment times so as to minimize the chances of reasons other than insufficient financial incentives to affect their failure to collect the payment; and that they provide an email contact (or equivalent) in case of any issues preventing the subject from collecting their payment.

Still another problem for our interpretation could be that collectors are not necessarily taking the experiment seriously. This cannot be ruled out entirely, and in this sense, when we talk of the restricted dataset, it is just a shortcut for subjects who are likely (but of course not certain) to satisfy dominance. While not perfect, our procedure is one step forward, however, towards a better control of whether dominance is satisfied.

A different story for why subjects may not have claimed the money is that they were not altruistic towards Oxfam but rather towards the experimenter. We are aware of two studies that clearly and explicitly test for altruism towards the experimenter, and neither finds any evidence for it [33,34]. That said, and even were this story to apply to some degree, it would only provide a different reason–and one common to most non zero-sum economic experiments routinely published in economics journals–for why drop-out should be excluded.

### Charitable giving

The relative amounts donated in our experiment (about 5–6% of the endowment) were comparable in size with what is actually donated in the real world (e.g. 1–3% in the UK [35], and 5.63% in the US [36]) but smaller than those observed in other (field) experiments on charitable giving [37]. The latter difference might in part be caused by the fact that in our study participants had to earn their endowment instead of just receiving it.

Testing whether different reminder frequencies could affect the amount donated and the probability of making a donation revealed that it could be beneficial for charitable organizations to send email reminders. We did not find any evidence that reminders increased the amount donated on the full dataset. However, in the restricted dataset, other things being equal, we found that both monthly and weekly reminders had a significant and substantial positive effect on the amount donated. The probability of making a donation increased by around 10% as a result of reminders. The likely reason for this could be that not reminding people could simply make them forget about their opportunity to donate to charity (‘out of sight, out of mind’).

We did not find significant differences between the effects of monthly and weekly reminders. Weekly reminders do not seem to be consistently better than monthly reminders, possibly because monthly reminders are enough to avoid ‘out of sight, out of mind’, and/or possibly because some participants are used to ignore too frequent repeat emails as equivalent of spam. Future research could look outside the range of one week to one month. It is entirely possible that reminders more frequent than 1 week, e.g. daily reminders, may backfire, which may help us understand where people draw the line between just receiving or tolerating information and being spammed. It is also worth mentioning that such a line might be quite different for a typical student population which is more used to receiving many emails frequently than the general population. As it is common recommendation for actual charities to send out ‘reminders’ three to six times per year, i.e. every two to four months [38–40], it may also be interesting to see how much less frequent a reminder could be while remaining effective, given the obvious social desire to reduce email traffic.

In our three months duration experiment we did not find evidence for an effect caused by the method of payment, i.e. whether participants could make one-off donations or could set up standing orders. The hypothesis of finding a difference between the two methods of payment was based on previous research which found that people might stick to their default, i.e. are less likely to cancel a standing order once it is up and running (status quo bias, [25]). However, for rational decision makers the method of payment should not make a difference anyway. Two things might have nudged our participants to more rational decisions than the general public would potentially have taken in their daily reality. First, although the experiment lasted three months and the experimental earnings were spread across three monthly salaries, three months are still a finite horizon that terminates any standing orders in the near foreseeable future. Thus participants might, quite rationally, have decided on the amount they wanted to donate and then, dependent on their method of payment, either donated this amount in a one-off transaction or set up a standing order that transfers one third of the planned amount each month, in the end resulting in exactly the same amount donated. That is, we find that at the very least three months seem not to be enough to make donors fall prey to inertia effects. Second, in the questionnaire before receiving their final payment, many participants mentioned that they were living on a tight budget and they needed to keep track of their financial activities very precisely. Suffering from financial pressure might draw more attention to financial issues in general which could make is less likely to fall for a status quo bias in financial matters or to ‘forget’ about an initially set up standing order.

Of course, one limitation of our experiment is that in the natural world donations occur over an extended period rather than just three months. That said, any experimental research translates real world situations into stylized settings, and a three months duration is longer than that employed in most economic experiments, including those on charitable giving. Obviously, it is an interesting avenue for future experimental research to extend the duration to even longer time horizons, e.g. one year.

## Conclusion

We do find a key difference in experimental results depending on whether the sample is restricted to participants that drop out or not. Specifically, reminders raise charitable giving if we focus on the restricted dataset, but not otherwise. Regardless of the dataset, we do not instead find that weekly reminders work significantly better than monthly reminders in raising charitable giving, and in our three months duration experiment the use of standing orders in place of one off donations is equally ineffective.

Screening out drop-outs is arguably a way of reducing noise. Our best interpretation of this is in terms of dominance. For this interpretation of drop outs to make sense, it requires subjects to know at the beginning that payment will take place in a given time period. It also requires researchers to provide flexibility in the payment times so as to minimize the chances of reasons other than a violation of dominance to affect their failure to collect the payment; and to provide an email contact (or equivalent) in case of any issues preventing the subject from collecting their payment.

## Supporting Information

S1 File Supplementary information. (DOCX)
