# Supplementary material for: On Reminder Effects, Drop-Outs and Dominance: Evidence from an Online Experiment on Charitable Giving
Source: PLoS One. 2015 Aug 7;10(8):e0134705. doi: 10.1371/journal.pone.0134705 (PMC4529084; doi:10.1371/journal.pone.0134705)
Supplement: S1 File — (DOCX) [file pone.0134705.s001.docx]

# S1 File. Supplementary information.

## Procedural details

During the period of the experiment, monthly reminders were sent every third Monday of a month, i.e. on February 18^th^, March 18^th^ and April 15^th^. Weekly reminders were sent every Monday, irrespective how many weeks a month had. Hence, weekly reminders were sent on February 18^th^. 25^th^, March 4^th^, 11^th^, 18^th^, 25^th^, April 1^st^, 8^th^, 15^th^, 22^nd^, 29^th^ and May 6^th^ and 13^th^ resulting in 13 weekly reminders. We chose to send ‘monthly’ reminders every fourth week (instead of sticking to a specific date, e.g. the 15^th^ of each month) as we were interested in executing exact control over the reminder intensity (every week vs. every fourth week). We do not believe that not fixing a specific date (e.g. the 15^th^ of each month) could have any detrimental consequences regarding our research design, especially as the used dates for monthly reminders were saliently communicated to the participants in the instructions as well as on the online experimental platform. Standing orders, if any, were executed on the 14^th^ of March, 14^th^ of April and the 14^th^ of May, i.e. any donations made via standing order were deducted from the participants’ online accounts on these dates.

## Pre-lab questionnaire

*Instruction*

*Below you will find a list of statements. Please read each statement carefully and decide if that statement describes you or not. If it describes you, check the word "true"; if not, check the word "false".*

*1. I sometimes litter.*

*2. I always admit my mistakes openly and face the potential negative consequences.*

*3. In traffic I am always polite and considerate of others.*

*4. I have tried illegal drugs (for example, marijuana, cocaine, etc.).*

*5. I always accept others' opinions, even when they don't agree with my own.*

*6. I take out my bad moods on others now and then.*

*7. There has been an occasion when I took advantage of someone else.*

*8. In conversations I always listen attentively and let others finish their sentences.*

*9. I never hesitate to help someone in case of emergency.*

*10. When I have made a promise, I keep it--no ifs, ands or buts.*

*11. I occasionally speak badly of others behind their back.*

*12. I would never live off other people.*

*13. I always stay friendly and courteous with other people, even when I am stressed out.*

*14. During arguments I always stay objective and matter-of-fact.*

*15. There has been at least one occasion when I failed to return an item that I borrowed.*

*16. I always eat a healthy diet.*

*17. Sometimes I only help because I expect something in return.*

***[This Note was not part of the instructions. Answer categories are "true" (1) and "false" (0). Items 1, 4, 6, 7, 11, 15, and 17 are reverse keyed. Item 4 was deleted from the final version of the SDS-17.]***

**** next page ****

- *Would you consider yourself to be a very organized person? [7-item Likert]*
- *Would you consider yourself to be a very busy person? [7-item Likert]*

**** next page ****

- *How many emails do you on average receive per day? [open]*
- *How many of those would you consider worth reading? [open]*

**** next page ****

- *What is your current age (in years)? [open]*
- *What is your gender? [Male, Female]*
- *What is your main Field of Study? [open]*
- *What is your nationality? [open]*

**** next page ****

- *What is your first name? [open]*
- *What is your surname? [open]*
- *What is your campus card number [open, number]*
- *What is your Email-address? [open]*

## Materials used during the lab sessions

### Consent form

Before starting with the lab session, each participant was asked to fill a consent form.

***Consent Form***

*I declare to have volunteered to participate in this experiment. I understand and accept that I may regularly receive emails throughout the duration of this experiment (3 months).*

*Furthermore, I understand that the reports of this experiment will not identify me, and that my participation in the experiment will not affect my academic standing at the University.*

*Name of Participant:*

*Campus Card Number:*

*Date:*

*Signature:*

*To be completed by the experimenter:*

*Session:*

*Date:*

### Task instructions

*In the following you will be provided with a set of different 5x5 matrices similar to the one depicted below that contain zeros and ones. The amount of zeros and ones as well as their distribution within the matrix will be different for every matrix.*

*You are asked to count how many “1” are in the matrix and type in this number in the text box displayed below the matrix.*

*Example*

| *0* | *1* | *1* | *0* | *0* |
| --- | --- | --- | --- | --- |
| *1* | *0* | *0* | *0* | *1* |
| *0* | *0* | *1* | *1* | *1* |
| *0* | *1* | *0* | *1* | *1* |
| *1* | *1* | *1* | *0* | *0* |

*Number of 1s:*

*Submit count and proceed to next matrix*

*Once you have counted the number of “1s” and typed your count in the text box provided, you can submit your count and proceed to the next matrix.*

*Every time you typed in the correct number of “1s”, you are rewarded with a token. You will have 10 minutes time to gather as many tokens as possible. Please note that the programme will stop automatically after 10 minutes.*

*Example:
Considering the matrix displayed above, the correct answer would be 13 (in fact there are 13 “1s” and 12 “0s”). If you typed in “13” and clicked the “Submit count and proceed to next matrix” button you would receive a token.*

*Payment:
If your final amount of tokens is less than 15, you will receive £2 participation fee paid in cash directly after this session.*

*If you could gather at least 15 tokens, you will receive £2 pounds participation fee directly after this session and £5 pounds monthly salary paid to a personal online account over the next 3 months. In fact, you would receive £5 by tomorrow, £5 on March 15^th^ and another £5 on April 15^th^ (adding up to £15 in total) on your online account. After three months, the balance of the experimental account will be paid in cash on May 15^th^ (or whenever collecting the money is convenient for you in the following four weeks thereafter). Exact collection dates and times will be provided by email on May 15^th^.*

***Should you have any questions about the task or the method of payment, please contact the experimenter!***

*You will be able to check your online balance at any time via an online information portal that will be explained in more detail shortly.*

*Please raise your hand once you read and understood the above instructions.*

### Check for understanding

The following questionnaire was used to check whether participants understood the experimental set up. Participants could only continue once they answered all questions correctly.

- *How do you get to know the URL (web-address) of the online platform?*
  - *By following the link that will be provided in the confirmation email*
  - *By searching for it on the CBESS website*
  - *By asking the experimenter*
- *What will you be able to do on the website?*
  - *Register for further experiments*
  - *Donate to charity*
  - *Check account balance*
  - *Send email to the experimenter*
- *How much money could you donate every month?*
  - *Between 0 and 5 pounds*
  - *Between 0 and 10 pounds*
  - *Between 0 and 15 pounds*
- *What is Oxfam*
  - *An international company*
  - *An international charity against poverty*
  - *An international charity against environmental pollution*
- *If you chose to donate to Oxfam, how will the money you donated actually reach Oxfam?*
  - *The experimenters will transfer your donations to Oxfam immediately after you made them*
  - *The experimenters will transfer the cumulative amount of donations to Oxfam at the end of May*
- *How much will you get when the experiment ends?*
  - *15 pounds, independent from any charitable donations made on the online platform*
  - *15 pounds minus any charitable donations made on the online platform*
  - *15 pounds minus double the amount of any charitable donations made on the online platform*
  - *15 pounds minus 67% of the amount of any charitable donations made on the online platform*
- *How will you receive your earnings (minus your donations, if applicable)*
  - *Collect them personally on May 16^th^ or thereafter if convenient, at a location to be specified in an email the experimenter will send to remind you of the payment date*
  - *Receive a bank transfer on May 16th payable to an account to be provided via the online platform*
  - *Collect them personally on May 16^th^ at 11:00am in room 3.68 in the building Arts 2*

### Procrastination inventory

- *I delay making decision until it’s too late.*
- *Even after I make a decision I delay acting upon it.*
- *I waste a lot of time on trivial matters before getting to the final decisions.*
- *In preparation for some deadlines, I often waste time by doing other things.*
- *Even jobs that require little else except sitting down and doing them, I find that they seldom get done for days.*
- *I am continually saying “I’ll do it tomorrow”.*
- *I generally delay before starting on work I have to do.*
- *I find myself running out of time.*
- *I don’t get things done on time.*
- *I am not very good at meeting deadlines.*
- *Putting things off till the last minute has cost me money in the past.*

## Questionnaire at the end of the experiment (before collection on money)

*Please answer the following questions.
Your answers will be handled absolutely anonymously.*

1. *You may have received reminders on your opportunities regarding the online platform during the last three months. How often did you receive such emails on average?*

- *Never or almost never*
- *About monthly*
- *About weekly*

1. *How often did you check your emails during the Easter break?*

- *Never or almost never*
- *Once a week*
- *At least twice a week*

1. *Do you remember how much you donated?*

- *I do not remember.*
- *I donated (approximately) ____ pounds in total (Please fill in your amount).*

1. *Why did you choose to donate (or not) the amount you did? Please describe briefly.*

## Email texts

### Reminder emails

The text of the email reminders was exactly the same across all reminder treatments (weekly, monthly). However, we used different texts before and after a participant made his/her first donation.

#### Before a participant made his/her first donation

*Dear #name#,*

*Did you know that your donation could make an important difference? By supporting Oxfam you would help reducing global poverty.*

*If you would like to support Oxfam, please follow http://ueaexperiments.org/ifeocg/online_phase.php to log on to our online information platform and make a donation.*

*By logging in you would also be able to check your account balance.*

#### After a participant made his/her first donation

*Dear #name#,*

*Thank you for supporting Oxfam. Your contribution will make an important difference.*

*If you would like to continue helping to reduce global poverty, please make a donation by following http://ueaexperiments.org/ifeocg/online_phase.php. By logging in you would also be able to check your account balance.*

### Further email texts used during the experiment

#### Invitation email to participate in the experiment

*Hello NAME,*

*We would like to invite you to participate in an upcoming decision-making experiment at the Centre for Behavioural and Experimental Social Science.*

*IMPORTANT NOTE: You can only take part in this experiment after finishing the online questionnaire on https://www.surveymonkey.com/s/NYM6JVV.*

*After you finished the questionnaire you can participate in one of the sessions below. Sessions will last at most 30 minutes. If you arrive on time, but the session is fully booked, you will receive a participation fee in the amount of £2.*

*The sessions are scheduled for the following times:*

*--- Session times ---*

*(Note that you may sign up for at most one of these sessions.)*

*The exact amount you can earn from participating solely depends on the decisions you make. By participating in this experiment you can earn up to £17.*

*Please note that you will receive the participation fee of £2 directly after the participating in the 30 minute session. The rest of your experimental earnings will not be paid in cash immediately after the session but will be transferred to an online experimental account as a monthly salary over a period of three months. At the end of this period, the balance of the experimental account will be paid in cash on May 15^th^ (or whenever collecting the money is convenient for you in the following four weeks thereafter). Exact collection dates and times will be provided in due course.*

*You should make sure to have internet access at least once a week over the next three months.*

*AFTER COMPLETING the survey, in order to sign up for one of the sessions mentioned above, go to the following webpage:*

*http://cbess.webapp3.uea.ac.uk/public/participant_show.php?p=cdlDfyJMZwQJc*

*Please remember that completing the questionnaire (first link above) is required in order to participate in one of the experimental sessions (second link above).*

*Each session is designed for a fixed number of participants. Those who arrive earlier will have priority, so please plan to arrive early to avoid disappointment!*

*Sessions begin promptly at the time listed. If you arrive late, we regret we will not be able to offer you a participation fee or an opportunity to take part in the session.*

*Please respect your fellow participants by being prompt.*

#### Pre-lab questionnaire reminder

Participants who registered for the experiments after receiving a standard CBESS invitation email had one week’s time to fill an online questionnaire before the lab session. The following email was used to remind them that on the pre-lab online questionnaire.

*Dear student,*

*You recently registered to participate in a CBESS experiment (name: 2013.003, sessions from 11/02/2013 to 13/02/2013).*

*Please note that you can only take part in this experiment after finishing the online questionnaire on http://www.ueaexperiments.org/ifeocg/prelab_survey.php*

*Please make sure to fill in the questionnaire before Monday, 11/02/2013. Otherwise, you cannot participate in the above experiment and you will also not receive a show up fee.*

#### Informing participants who failed to fill the pre-lab questionnaire in time

Participants who failed filling the pre-lab questionnaire until one day before the first lab session were informed that they were not eligible to participate in this experiment any further.

*Dear student,*

*You signed up for an experiment that required you to complete an online questionnaire BEFORE signing up. You are receiving this email because according to our records you didn't complete the questionnaire. Therefore, we are sorry to inform you that you will be removed from the participants list for the session you signed up for (this will not affect your records in any manner).*

*If you did complete the questionnaire, please contact us by email as soon as possible. Provide us the campus card number you wrote in the online questionnaire, it could be that it doesn't match the number you provided when registering in our recruitment system and that is the reason why we can't find you.*

#### Confirmation of registration to the online information platform

During the lab session participants registered for the ‘online information platform’. This registration was confirmed by the following email which all participants received after the lab part was finished. The parts #name#, #password# and #outpost_account# were replaced by the participant’s name, personally set password and the email account which later on was used to send reminder emails, if any.

*Dear #name#,*

*Thank you very much for registering to our online information platform. By following http://ueaexperiments.org/ifeocg/online_phase.php you could log on to the platform at any time. Remind that by doing so you could check your account balance and donate to charity. In order to successfully log on you need to identify yourself with your personal username and password. Your username simply is the email address you were receiving this email. Your password is >>#password#<<.*

*Please make sure that your spam filter is set to allow emails from #outpost_account#.*

*Should you have any problems logging in or any other questions please contact us at info@ueaexperiments.org*

#### Final email: collection of payment reminder

After three months, all participants received the following email to remind them on the approaching payment date and the concrete location and time. #name# and #time# were replaced by the participant’s name and his/her timeslot which was randomly assigned to each participant and was always between 13:00 and 17:00 hours on the payment day (15^th^ of May).

*Dear #name#,*

*The online part of the experiment you participated in ended. You can no longer access the online information platform, i.e. you can no longer check your balance or make donations to Oxfam.*

*You can collect your final payment (15 pounds minus the sum of your donations to Oxfam) tomorrow, on Wednesday, 15th of May at #time# in room 3.27 in the building Arts 2. Please make sure to have your campus card with you for identification.*

*We strongly advise you to arrive at exactly #time# to avoid unnecessary waiting times. However, if you are not able to collect your money at your specific collection time, you can collect your payment at any time between 13:00 and 17:00 o'clock on Wednesday, 15th of May.*

*If you are not able to collect your money on the 15th of May at all, you can collect it on Friday, 17th of May between 14:00 and 16:00 o'clock in the room 3.27 in the building Arts 2.*

*If you are not able to collect your money on either May 15th or May 17th, please write us an email to info@ueaexperiments.org.*

*Thank you for participating in our experiment!*

#### Flexible collection time arrangements

It was very important to our design that participants could collect their payment even if they were not able to use one of the official payment days for doing so. We were therefore flexible with regards to collection times and arrangements. Below we reproduce an anonymized email conversation that exemplifies how we dealt with individual circumstances of participants. In total 47 participants sent us 89 emails of this kind (counting each email received, i.e. counting the first approach and any further response separately).

Email received by participant on the 13^th^ of June 2013:

*Dear Sir/ Madam*

*I am really sorry I could not make it these days to come and collect the money. I am currently not in Norwich. Is it possible to give the 15 pounds to my friend xxx when she come in to collect hers?*

*My name: xxx*

*Student No: xxx*

*Many Thanks*

*xxx*

Response sent by the experimenters on the 13^th^ of June 2013:

*Dear xxx,*

*Yes that is possible if you filled and returned the attached questionnaire by the time your friend collects her money. Without the filled questionnaire, there cannot be any payment.*

*Best wishes*

Response sent by participant on the 14^th^ of June 2013:

*Dear Sir/ Madam*

*Thank you very much for the reply. I have attached the filled questionnaire with this mail.*

*xxx*
